# *Exophiala dermatitidis* pneumonia with bronchiectasis required prolonged voriconazole treatment

**DOI:** 10.1002/rcr2.783

**Published:** 2021-05-25

**Authors:** Ryo Sekiguchi, Naohisa Urabe, Susumu Sakamoto, Masakazu Sasaki, Sakae Homma, Kazuma Kishi

**Affiliations:** ^1^ Department of Respiratory Medicine Toho University Omori Medical Center Tokyo Japan; ^2^ Department of Clinical Laboratory Toho University Omori Medical Center Tokyo Japan; ^3^ Department of Advanced and Integrated Interstitial Lung Disease Research Toho University School of Medicine Tokyo Japan

**Keywords:** Bronchiectasis, *Exophiala dermatitidis*, non‐cystic fibrosis, voriconazole

## Abstract

*Exophiala dermatitidis* is a black fungus that rarely causes respiratory infection. We report a case of *E*. *dermatitidis* pneumonia with bronchiectasis that relapsed after 11 months of voriconazole (VRCZ) treatment in a rheumatoid arthritis (RA) patient with bronchiectasis. A 65‐year‐old woman with RA and abnormal findings on chest radiography was referred for assessment of chronic cough and increased sputum production. She underwent bronchoscopy, and *E*. *dermatitidis* was identified from bronchoalveolar lavage fluid (BALF). *Exophiala dermatitidis* chronic lower respiratory tract infection and pneumonia were diagnosed. Although her condition improved after 11 months of VRCZ treatment, chest computed tomography (CT) images showed worsening at five months after the cessation of VRCZ treatment and *E*. *dermatitidis* was again detected in BALF. Re‐administration of VRCZ for two years improved symptoms and chest CT images, and her condition is currently stable. In patients with bronchiectasis, *E*. *dermatitidis* pneumonia might require prolonged antifungal treatment.

## Graphical abstract

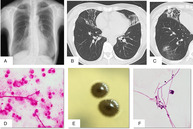

## Introduction

*Exophiala dermatitidis* is a black fungus that is distributed widely in the natural environment. *Exophiala dermatitidis* can cause skin and corneal infections, and pulmonary *E*. *dermatitidis* infection was reported in patients with and without cystic fibrosis (CF) bronchiectasis. The appropriate duration of antifungal therapy for pulmonary *E*. *dermatitidis* infections is unclear. We describe a case of *E*. *dermatitidis* pneumonia in a woman with bronchiectasis.

## Case Report

A 65‐year‐old woman with a history of dyslipidaemia and osteoporosis was referred to our hospital for assessment of cough of one month's duration, increased sputum production, and an abnormal chest X‐ray. Rheumatoid arthritis (RA) was diagnosed three weeks previously because of joint pain in four fingers and an anti‐cyclic citrullinated peptide antibody level of 31.8 U/mL. She was receiving bucillamine and had no history of smoking, recurring pneumonia, other infections, dust exposure, or allergies, and no family history.

On admission, her temperature was 36.4°C, pulse rate 74 beats/min, and respiratory rate was 18 breaths/min. Respiratory examination revealed clear breath sounds. Laboratory analysis revealed C‐reactive protein level of 0.8 mg/dL (normal <0.3), white blood cell count of 5400 cells/μL, and an immunoglobulin G level of 1586 mg/dL. Serum β‐d glucan level was 11.4 pg/mL (normal <10), and serum aspergillus galactomannan antigen, aspergillus antibody, HIV, and interferon‐gamma release assay were negative.

**Figure 1:**
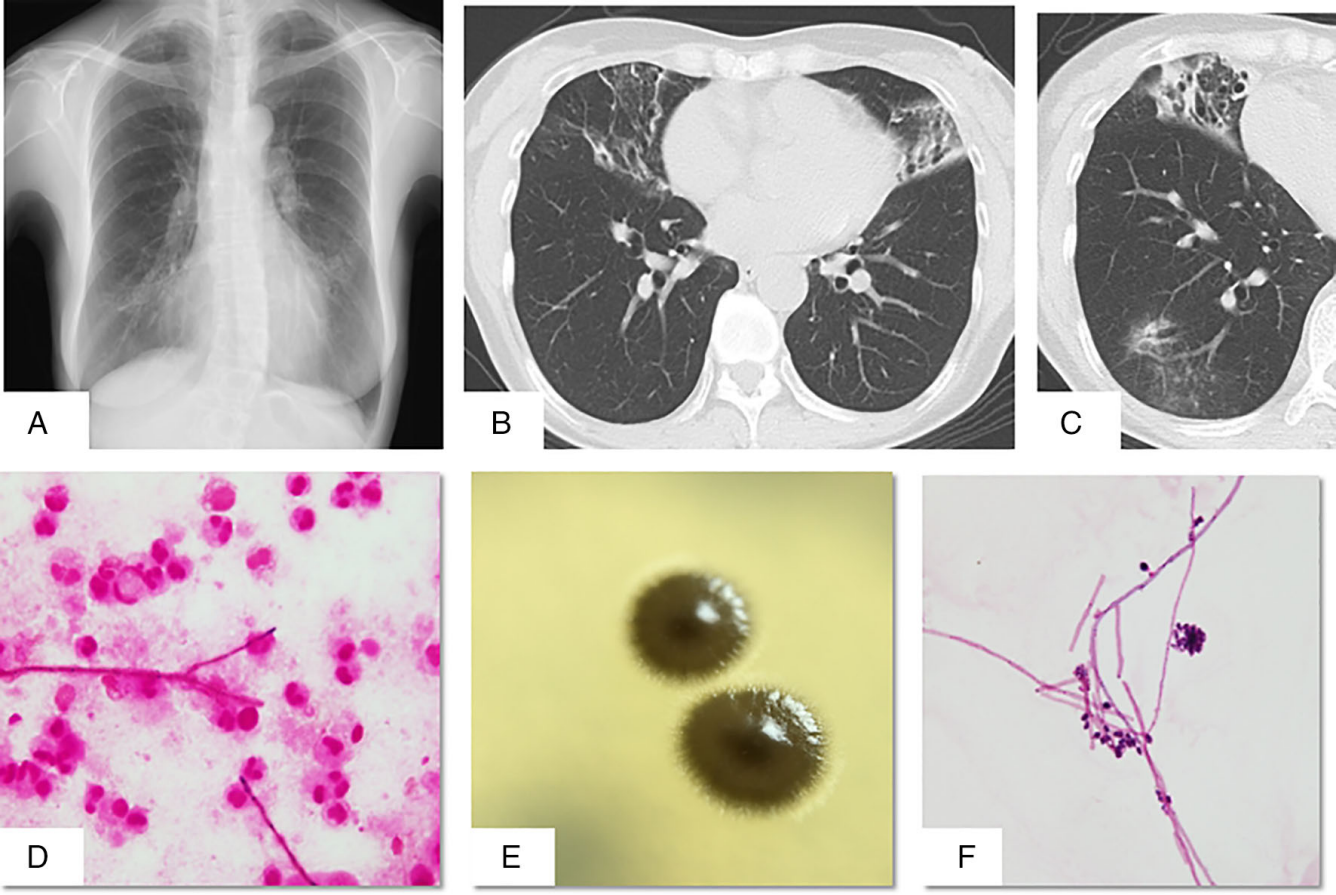
(A) Chest radiograph showing an infiltrative shadow in the mediastinum of the right lower lung field and ground‐glass shadows in the lower lung field bilaterally. (B) Chest computed tomography (CT) images: right middle lobe and lingula, invasive lesion, and bronchiectasis. (C) Granular lesion and ground‐glass shadow in the lower right lobe (lateral basal and posterior basal). (D) Gram stain of bronchial lavage fluid revealed filamentous fungi (100× magnification). (E) A melted chocolate‐like, olive‐brown viscous colony on Sabouraud agar. (F) Gram stain of bronchial lavage fluid showing filamentous fungi forming hyphae and conidia (1000× magnification).

A chest radiograph revealed infiltrate opacities bilaterally, predominantly in the lower lung (Fig. 1A). High‐resolution chest computed tomography (CT) revealed bilateral bronchiectasis and consolidation associated with centrilobular nodules in the right middle lobe and lingula (Fig. 1B). In addition, small nodular lesions and ground‐glass opacities were observed in the right lateral and posterior basal segments (Fig. 1C).

A sputum culture was negative. Bronchoscopy was done; Gram staining of bronchoalveolar lavage fluid (BALF) revealed filamentous fungi (Fig. 1D). BALF cultures using Sabouraud agar yielded olive‐brown viscous colonies at seven days (Fig. 1E). Hyphal and conidial formation were observed in the colonies (Fig. 1F). Matrix‐assisted laser desorption ionization‐time‐of‐flight mass spectrometry (MALDI‐TOF MS) identified the fungus as *E*. *dermatitidis*. Bacteria and mycobacteria were not detected in BALF. Polymerase chain reaction (PCR) assay for *Mycobacterium tuberculosis* and *Mycobacterium avium*–*intracellulare* was negative. *Exophiala dermatitidis* respiratory infection was diagnosed based on clinical, radiological manifestation, and microbiological results. Minimum inhibitory concentrations (MICs) were itraconazole (ITCZ) 0.25 μg/mL, voriconazole (VRCZ) 0.12 μg/mL, amphotericin B (AMPH‐B) 1 μg/mL, and micafungin (MCFG) >16 μg/mL.

**Figure 2:**
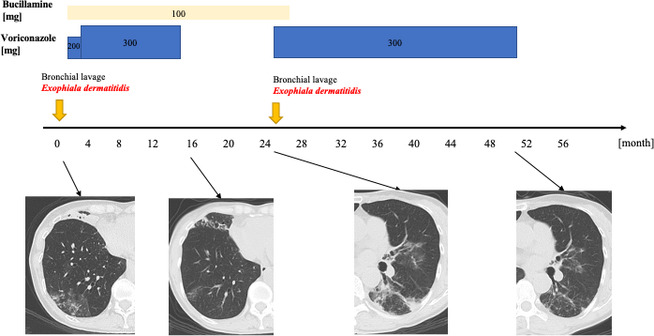
Clinical course of the present patient from the initial visit.

Oral VRCZ 200 mg/day was started. The dose was increased to 300 mg/day after 20 days and adjusted according to blood concentration (1.0–4.5 μg/mL). Chest CT images showed that lesions in the right middle and lower lobes and lingula had almost disappeared after 11 months of treatment. Cough and sputum improved and VRCZ treatment was discontinued. We recommended bronchoscopy to confirm a negative *E*. *dermatitidis* result, but she declined. Her productive cough and chest CT images worsened at five months after treatment discontinuation, and *E*. *dermatitidis* was again detected in BALF. Re‐administration of VRCZ for two years improved her symptoms and chest CT images, and VRCZ treatment was terminated. Her disease status remains stable (Fig. 2).

## Discussion

Although studies have reported *E*. *dermatitidis* infection and colonization in CF patients, only four cases of *E*. *dermatitidis* pneumonia have been reported in non‐CF patients [1, 2, 3, 4]. All four were female: three had idiopathic bronchiectasis [1, 2, 3] and one had a nodular lesion that was difficult to distinguish from lung cancer [4]. Our patient had RA, which might have been the cause of bronchiectasis. *Exophiala dermatitidis* may have settled in areas affected by bronchiectasis.

Because CF is rare in Japan, genetic testing for CF is unusual. In our patient, the absence of respiratory symptoms since childhood and presence of a main lesion on chest CT in the right middle lobe and lingula were consistent with non‐CF bronchiectasis.

*Exophiala dermatitidis* infection is rare, even in non‐immunocompromised patients [1, 2, 3, 4]. Although our patient was not immunodeficient, she had respiratory symptoms, bronchiectasis, and pneumonia findings on chest CT, and *E*. *dermatitidis* was the only pathogen isolated. We thus diagnosed chronic lower respiratory tract infection and pneumonia caused by *E*. *dermatitidis*.

*Exophiala dermatitidis* is sensitive to terbinafine, VRCZ, ITCZ, fluconazole, AMPH‐B, and caspofungin [5]. In non‐CF cases, some studies reported that ITCZ and VRCZ were effective for pulmonary *E*. *dermatitidis* infection. Regardless of CF status, appropriate duration of treatment for pulmonary *E*. *dermatitidis* infection is unclear. In a patient without CF, chest CT images and clinical symptoms improved after five months of ITCZ treatment, but relapse occurred two weeks after treatment cessation. ITCZ therapy was then restarted and continued for seven months [1]. The patient's clinical course suggested that more than one year of ITCZ treatment may be necessary to treat pulmonary *E*. *dermatitidis* infection in patients without CF. However, four months of VRCZ monotherapy was effective for pulmonary *E*. *dermatitidis* infection in patients without CF [4].

Our patient relapsed after 11 months of medical treatment. Retreatment for two years was effective, and no relapse has developed so far. We hypothesize that RA and bucillamine diminished immune response to *E*. *dermatitidis* and decreased local clearance. Bucillamine is an anti‐rheumatic drug in Japan used to treat early RA and has immunomodulatory effects. In addition, we suspect it is difficult to cure fungal chronic lower respiratory tract infections with bronchiectasis.

Although the long‐term outcomes of pulmonary *E*. *dermatitidis* infection are unclear, appropriate antifungal treatment is likely beneficial. Recurrence can occur after discontinuing treatment in patients with underlying conditions such as bronchiectasis. Our experience suggests that prolonged VRCZ therapy may be needed to treat pulmonary *E*. *dermatitidis* infection in bronchiectasis.

### Disclosure Statement

Appropriate written informed consent was obtained for publication of this case report and accompanying images.
